# Developing an Intervention to Reduce Sedentary Behavior: Assisted Living Resident Feedback

**DOI:** 10.1093/geroni/igaa057.1227

**Published:** 2020-12-16

**Authors:** Katelyn Webster, Janet Larson

**Affiliations:** University of Michigan School of Nursing, Ann Arbor, Michigan, United States

## Abstract

Older adults in assisted living (AL) tend to be highly sedentary, which increases their risk of functional decline and frailty. Reducing sedentary behavior (SB) and replacing it with light physical activity (LPA) could have important implications for maintenance of functional abilities. The purpose of this qualitative study was to gather feedback from AL residents on a proposed exercise-specific self-efficacy enhancing intervention designed to promote LPA and reduce SB. We conducted one-on-one semi-structured interviews at four AL facilities with 20 residents ages 65-99 (mean age 83.1; 60% women). They were presented with the proposed intervention and were asked questions to inform the development and modification of the intervention. Data were analyzed with a thematic analysis approach. Specific recommendations included short intervention sessions of one hour and scheduled in the morning. Many residents thought a 16 week intervention was too long. We identified broader themes, including concerns about how the intervention would work for residents with a wide range of abilities and concerns about safety. They suggested that exercises will need to be highly modifiable. Most residents recommended framing the intervention message as increasing LPA rather than decreasing SB, because it would be more positive. All except one said they would want to participate in the intervention, but they were unsure how many other residents would want to be more active. The resident feedback and suggestions will guide development of the intervention and are important for increasing the probability that a future feasibility and acceptability trial of the intervention will be successful.

